# *Citrus aurantifolia* essential oil: antiaging potential through integrated *in vitro* and *in silico* studies

**DOI:** 10.3389/fchem.2025.1577871

**Published:** 2025-08-01

**Authors:** Yasmin A. Elkhawas, Mahmoud A. El Hassab, Omkulthom Al kamaly, Ahmed T. Negmeldin, Taghreed A. Majrashi, Wagdy M. Eldehna, Nada M. Mostafa

**Affiliations:** ^1^ Department of Pharmacognosy and Medicinal Plants, Faculty of Pharmacy, Future University in Egypt, Cairo, Egypt; ^2^ Medicinal Chemistry Department, Faculty of Pharmacy, King Salman International University, Ras-Sedr, South Sinai, Egypt; ^3^ Department of Pharmaceutical Sciences, College of Pharmacy, Princess Nourah Bint Abdulrahman University, Riyadh, Saudi Arabia; ^4^ Department of Pharmaceutical Sciences, College of Pharmacy and Thumbay Research Institute for Precision Medicine, Gulf Medical University, Ajman, United Arab Emirates; ^5^ Department of Pharmaceutical Organic Chemistry, Faculty of Pharmacy, Cairo University, Cairo, Egypt; ^6^ Department of Pharmacognosy, College of Pharmacy, King Khalid University, Asir, Saudi Arabia; ^7^ Department of Pharmaceutical Chemistry, Faculty of Pharmacy, Kafrelsheikh University, Kafrelsheikh, Egypt; ^8^ Department of Pharmacognosy, Faculty of Pharmacy, Ain Shams University, Cairo, Egypt

**Keywords:** 2,2′-azino-bis(3-ethylbenzothiazoline-6-sulfonic acid) (ABTS), antiaging, anticollagenase, anti-elastase, *Citrus aurantifolia*, GC/MS, key lime

## Abstract

**Introduction:**

Natural skincare products and cosmetic preparations have gained popularity among consumers in recent years, prompting cosmetic companies to develop more natural offerings. These products often incorporate plant extracts known for their anti-aging, anti-wrinkle, and depigmentation properties.

**Methods:**

Using gas chromatography-mass spectrometry, this study examined the volatile compounds in both fresh and dry *Citrus aurantifolia* (key lime) fruit essential oils. The oils’ anti-aging and antioxidant activities were assessed through *in vitro* anti-collagenase and anti-elastase assays.

**Results:**

*In vitro* analysis revealed good inhibition of elastase and collagenase enzymes by fresh key lime essential oil, with IC_50_ values of 145.02 and 63.97 μg/mL, respectively, compared to positive controls (daidzein for collagenase, piroxicam for elastase), in comparison to dry key lime oil (IC_50_ = 223.14 and 109.57 μg/mL, respectively). The antioxidant activity of the oils was evaluated using the ABTS (2,2’-azino-bis(3-ethylbenzothiazoline6-sulfonic acid)) radical scavenging assay. The fresh key lime oil demonstrated stronger antioxidant activity (37.76 ± 0.80 μM Trolox equivalent (TE)/g) compared to the dry key lime oil (27.76 ± 1.11 μM TE/g), suggesting that it retains more bioactive compounds essential for radical scavenging activity. Additionally, molecular docking was performed to analyze interactions between the main metabolites and the targeted enzymes active sites. Molecular docking analysis showed excellent binding scores for the three main metabolites.

**Conclusion:**

The anti-aging potential of fresh key lime essential oil may be attributed to its major compounds. These findings suggest that key lime essential oil could be a promising natural ingredient for anti-aging skincare formulations.

## 1 Introduction

*Citrus* species are among the most frequently eaten and extensively distributed fruits (Lin et al., 2019). The genus originated in Southeast Asia and is now found in various tropical and subtropical areas. Citrus fruits are categorized into several categories, including tangerines, mandarins, oranges, limes, lemons … etc. Because of their nutritional benefits and pleasant aroma and taste, these fruits are typically consumed whole fruits or as juices (Prommaban and Chaiyana, 2022). Citrus fruit (pulp, peel, and seed) provide potent bioactive metabolites as essential oils, phenolics, and flavonoids (Tavallali et al., 2021). These active metabolites displayed numerous biological properties as anti-inflammatory, antioxidant, anti-tumor, cardiovascular protective, and neuroprotective activities (Mahato et al., 2018). Essential oils are key secondary bioactive compounds (Rabie et al., 2023) that provide fragrances and are broadly employed in food additives, pharmaceuticals, and medicinal treatments (Prommaban and Chaiyana, 2022; El-Nashar et al., 2022). Citrus essential oils mostly consist of terpene hydrocarbons including monoterpenes, sesquiterpenes, and oxygenated derivatives (González-Mas et al., 2019). The most prevalent monoterpene hydrocarbon identified in various citrus essential oils was limonene (Mohammed et al., 2022).

*Citrus aurantifolia* belongs to the citrus genus, which is planted mainly in warm regions such as Egypt, Mexico, the Far east, and India (Spadaro et al., 2012). *C. aurantifolia* is a green colour fruit with smooth and thin skin and acidic juicy flesh with a defined odour and taste (Lin et al., 2019). The essential oil and juice are the major economic products of *C. aurantifolia* (Spadaro et al., 2012). Its essential oil constitutes mainly limonene, terpinen-4-ol, α-terpineol, β-pinene, eucalyptus, 1,4-cineole, p-cymene, geranial, β- bisabolene, and neral, which show various pharmacological activities as antioxidant, hypolipidemic, antidiabetic, anti-inflammatory, anticarcinogenic, antifungal, and antidepressant properties (Lin et al., 2019). Aside from these reported biological activities, anti-aging effects on the skin were rarely investigated.

Most of the research done on the *C. aurantifolia* was concerned with the analysis of the essential oils derived from the fruits and leaves (Spadaro et al., 2012; Jain et al., 2020) with significant attention on their antioxidant, antimicrobial, and anticancer activities (Naeini et al., 2018; Boshtam et al., 2011; Patil et al., 2009) that are mostly related to their high monoterpenes and oxygenated monoterpenes (Prommaban and Chaiyana, 2022). We were interested in studying the anti-aging activity of *C. aurantifolia* essential oils due to its verified antioxidant properties. Particularly, we planned to explore from a comparative point of view which oils, fresh or dried key lime oil, have the ability to inhibit collagenase and elastase enzymes to possibly use them as natural ingredients in managing skin age-related issues.

Skin ageing and wrinkles are significant issues of human skin that can be influenced by intrinsic factors (for example, genotype, endocrine metabolism, or level of hormones) and/or external factors (for example, ultraviolet radiation, nutrition, stress, and pollution) (Eun et al., 2020). Exposure of the skin to these external factors causes an increase in the enzymes (for example, collagenase and elastase) that participate in the ageing process. These enzymes initiate collagen and elastin breakdown, causing accelerated skin ageing which appears as wrinkles (Oulebsir et al., 2022).

Skin aging results from elastin and collagen diminution in the epidermal connective tissue, resulting in skin thinning and an increase in the wrinkles formation (Kim and Bae, 2023). The aging progression is related to the initiation of inflammatory pathways in the epidermis, usually indicated as “inflamm-aging” (Neves and Sousa-Victor, 2020). Ultraviolet radiation from the sun also plays an essential part in skin ageing and damage by promoting cytokines and intensifying the formation of free radicals (Lago and Puzzi, 2019). The cosmetics business is constantly researching active compounds to stop and minimize skin ageing, with cosmeceuticals being a critical part of anti-aging skin treatments (Shanbhag et al., 2019).

Essential oils are commonly used in skin care products and cosmetics because of their active metabolite with scented odor (Sharmeen et al., 2021). Many researchers have stated the usage of Citrus essential oils in cosmetics and pharmaceuticals (Prommaban and Chaiyana, 2022). Several research studies have revealed bioactive compounds and pharmacological properties of essential oils obtained from citrus flowers, leaves, and peels. However, research has been limited to the anti-ageing properties of key lime essential oil. Therefore, our study highlighted the chemical composition of both fresh and dried key lime essential oils, as well as their antioxidant activity and inhibitory effect on elastase and collagenase enzymes *in vitro*.

## 2 Results and discussion

### 2.1 Drying effect on the constituents and the yield of key lime essential oil

Both dried and fresh oil yields were 0.67% and 0.5% v/w, respectively. The essential oils have a distinguishing, faint yellow colour and a strong, lemon-scented odour. The constituents of both key lime essential oils are revealed in Table 1. Thirty-two and twenty-two compounds were separated and identified in fresh and dry key lime. In fresh key lime essential oil, 12 metabolites were monoterpenes, 10 oxygenated monoterpenes, and 10 sesquiterpene hydrocarbons, while in dried key lime essential oil, 11 metabolites were monoterpenes, 9 oxygenated monoterpenes, and 2 sesquiterpene hydrocarbons.

The major metabolite of the fresh key lime essential oil was D-limonene (42.02%), followed by β-pinene (10.88%), γ-terpinene (10.16%), and α-terpineol (7.84%). However, the main dry key lime essential oil compounds were D-limonene (72.64%), followed by γ-terpinene (15.67%). The shade drying method produced an alteration in the constituents, in which the percentage of monoterpenes hydrocarbons revealed a substantial increase, 76.42% in fresh oil to 96.24% in dry oil, whereas the oxygenated monoterpenes and sesquiterpene hydrocarbons decreased significantly (3.2%) in dry oil, in contrast to (13.51%) in fresh oil. This may be attributed to the isomerization of metabolites and short-chain alkenes (Elkhawas et al., 2022).

Furthermore, both α-terpineol, and 4-terpinenol, which have antioxidant, anti-inflammatory, and skin-regenerating characteristics (Chen et al., 2023; Be Tu and Tawata, 2015; Delgado-Adámez et al., 2017), dropped significantly from 7.84% to 1.71%, respectively, in fresh oil to 1.61% and 077%, respectively, in dry oil. Other oxygenated compounds, such as endo-borneol, fenchol, and γ-terpineol, were only present in the fresh sample. Sesquiterpene hydrocarbons, which made up 6.82% of fresh oil, were eradicated in dry oil (0.30%). Compounds including β-bisabolene, α-farnesene, and caryophyllene showed significant decreases.

D- Limonene is the prevalent chemotype found in essential oils produced from Citrus spp. various parts such as peels, fruits, flowers, and leaves (Lota et al., 2002). Lemes et al. reported that terpenes prevail in the essential oil collected from other *C. aurantifolia* specimens and that the compounds of the essential oil change greatly depending on the plant’s geographical locations and environmental factors (Lemes et al., 2018). Spadaro et al. explored the essential oil of key lime obtained from Italy and reported that D-limonene, γ-terpinene, and β-pinene presented the major compounds (Spadaro et al., 2012). Furthermore, Hong et al. stated that the major compounds identified in *C. aurantifolia* from South Korea were β-pinene and limonene (Hong et al., 2017).

Limonene, a lemon-fragrant monoterpene found in essential oils of citrus plants, is used in cosmetics, food, and drugs (Kim Y. W. et al., 2013). It was reported that it has various pharmacological properties such as anti-inflammatory, anticancer, antidiabetic, and antioxidant activities (Anandakumar et al., 2021; Secerli et al., 2023). Although previously reported data on *C. aurantifolia* fruits afforded about 46% of D-limonene (Liu et al., 2022). Yet, the percentage of limonene and γ-terpinene in our study was to some extent within range as previously reported data by Tavallali et al. (2021) and Sreepian et al. (2022). These variations were most likely due to different types of *C. aurantifolia* (variants, cultivars (Maria and Riccardo, 2020), and regions), and storage duration, harvest year (Gioffrè et al., 2020), season (Vekiari et al., 2002), climate change, and the extraction method (Sulzbach et al., 2021), all of which might change the chemical composition of essential oil (Elkhawas et al., 2023).

The essential oils of fresh and dry key lime fruits were predominantly D-limonene and γ-terpinene, which constitute 52.18% and 88.28%, respectively, of all components (Table 1). These compounds were stated to display antioxidant, anti-inflammatory, and anti-aging activities (Chen et al., 2023; Kumar et al., 2022). Moreover, these compounds can show a synergistic activity together (Boussaada et al., 2007). Kumar et al. have proved that limonene can shield keratinocytes from UVB-induced photoaging by preventing intracellular free radical formation, loss of skin barrier function, and cell death. Thus, it enables the skin to preserve its integrity and function (Kumar et al., 2022).

**TABLE 1:** Secondary metabolites of fresh and dry key lime’s essential oils are identified by GC-MS analysis.

Compound name	^a^Kovats index (RI) calculated	^b^Kovats index (RI)	Area% fresh	Area% dry	Identification
*Monoterpene hydrocarbon*					
α-Thujene	923	927	0.15	0.26	RI, MS
α-Pinene	929	939	2.79	1.79	RI, MS
Camphene	943	940	0.65	0.07	RI, MS
β-Pinene	971	981	10.88	1.33	RI, MS
β-Myrcene	988	993	1.65	2.27	RI, MS
D-Limonene	1031	1035	42.02	72.64	RI, MS
α-Phellandrene	1001	1003	0.49	0.22	RI, MS
α- Terpinene	1013	1017	--	0.55	RI, MS
γ-Terpinene	1057	1058	10.16	15.69	RI, MS
α-Terpinolene	1088	1090	4.36	1.35	RI, MS
α -Terpinyl acetate	1331	1330	--	0.07	RI, MS
O-Cymene	1022	1027	2.43	--	RI, MS
Trans-β-Ocimene	1035	1043	0.29	--	RI, MS
β -Ocimene	1045	1050	0.55	--	RI, MS
Monoterpene hydrocarbon total %			76.42%	96.24%	
*Oxygenated monoterpene*					
Linalool	1097	1094	0.44	0.14	RI, MS
β-Terpineol	1141	1159	0.56	0.13	RI, MS
4-Terpinenol	1174	1177	1.71	0.77	RI, MS
α-Terpineol	1188	1192	7.84	1.61	RI, MS
Trans-*p*-mentha-1(7),8-dien-2-ol	1185	1185	--	0.15	RI, MS
Cis-Dihydrocarvone	1194	1194	--	0.10	RI, MS
Cis-Carveol	1206	1214	--	0.11	RI, MS
Cis-*p*-mentha-1(7),8-dien-2-ol	1226	1231	--	0.08	RI, MS
(−)-Carvone	1242	1242	--	0.11	RI, MS
Fenchol	1110	1113	0.7	--	RI, MS
Endo-Borneol	1163	1165	0.78	--	RI, MS
1-Terpinenol	1131	1134	0.33	--	RI, MS
8-*p*-Cymenol	1184	1183	0.11	--	RI, MS
γ-Terpineol	1195	1199	0.78	--	RI, MS
Decanal	1202	1207	0.26	--	RI, MS
Oxygenated monoterpene total %			13.51%	3.2%	
*Sesquiterpene hydrocarbon*					
Caryophyllene	1418	1414	0.98	0.18	RI, MS
α-Farnesene	1504	1509	1.55	0.12	RI, MS
Elemene	1389	1382	0.07	--	RI, MS
Humulene	1453	1455	0.22	--	RI, MS
β-Santalene	1458	1457	0.66	--	RI, MS
α-Guaiene	1494	1490	0.1	--	RI, MS
Cis-α-Bisabolene	1499	1501	0.15	--	RI, MS
β-Bisabolene	1506	1509	2.89	--	RI, MS
Selina-3,7(11)-diene	1542	1542	0.12	--	RI, MS
Eudesma-4(14),11-diene	1486	1486	0.08	--	RI, MS
Sesquiterpene hydrocarbon total %			6.82%	0.30%	
Total percentage			96.75%	99.74%	

^a^ Calculated Kovats index (RI).

^b^ NIST, literature, and Adams Kovats index (RI). MS, mass spectral.

Several studies reported that various naturally occurring compounds, such as terpenes in essential oils, have anti-aging activity because of their antioxidant potentials (Cavinato et al., 2017), demonstrating that terpenes are capable of capturing free radicals and inhibiting UVB-induced photoaging (González-Burgos et al., 2012). Mohamed et al. stated that limonene exhibited antioxidant potential because of its ability to capture free radicals and has the ability to improve the effects of oxidative stress *in vitro* and *in vivo* (Mohammed et al., 2022). Furthermore, it was reported by Garg et al. that limonene exhibited high anti-inflammatory activity (Garg, 2017).

### 2.2 *In vitro* antiaging activity of fresh and dry *Citrus aurantifolia* essential oil

The antiaging capability of both *C. aurantifolia* essential oils was investigated *in vitro*. The findings showed promising activity of both essential oils towards collagenase and elastase enzymes and good antioxidant activity. Various quantities of the essential oils were tested and demonstrated dose-dependent inhibitory action against two enzymes (Table 2).

**TABLE 2:** IC_50_ values (µg/mL) for the anti-elastase and anti-collagenase activities for fresh and dry key lime essential oils.

Samples	IC_50_ (µg/mL)^*^	
	Elastase	Collagenase
Fresh essential oil	145.02 ± 6.37	63.97 ± 2.3
Dry essential oil	223.14 ± 9.39	109.57 ± 3.92
Daidzein	60.24 ± 2.64	-
Piroxicam	-	49.17 ± 1.77

*All results were made in triplicates and presented as mean ± SD.

Fresh key lime essential oil showed substantial anti-elastase and anti-collagenase potentials, IC_50_ = 145.02 ± 6.37 and 63.97 ± 2.3 µg/mL, respectively, close to that of the standard anti-aging drug with IC_50_ = 60.24 ± 2.64 and 49.17 ± 1.77 µg/mL, respectively as well as good antioxidant activity 37.76 ± 0.80 µM TE/g. Fresh key lime essential oil exhibits superior anti-elastase and anti-collagenase activities due to its higher content of bioactive oxygenated monoterpenes and sesquiterpenes. Key compounds like α-terpineol and 4-terpinenol enhance its antioxidant and anti-inflammatory effects, helping to protect collagen and elastin from degradation (Kim S. H. et al., 2013).

In this study, the compositional variations between fresh and dry key lime essential oils have important implications for their recognized anti-aging activity, especially since fresh oil contains more oxygenated monoterpenes and sesquiterpene hydrocarbons. According to Elgamal et al. sesquiterpenes have an important function as anti-oxidants, anti-inflammatory agents, and hence anti-aging properties (Elgamal et al., 2021).

While drying boosted D-limonene, the loss of oxygenated and sesquiterpene compounds likely lowers the oil’s efficiency in countering oxidative stress, inflammation, and collagen degradation, all of which contribute to skin ageing. As a result, the fresh key lime essential oil, with its more diversified bioactive composition, is predictable to have more anti-aging potential than the dry oil, making it a better choice for cosmetic and dermatological applications.

### 2.3 Antioxidant 2,2′-azino-bis (3-ethylbenzothiazoline-6-sulfonic acid (ABTS) assay

The scavenging capacity of both key lime essential oils was also evaluated using ABTS radical cation. The data suggests that the ABTS scavenging activity varied significantly between the essential oils. The ABTS radical scavenging activity of fresh key lime essential oil (37.76 ± 0.80 µM TE/g) was greater than that of dried key lime essential oil (27.76 ± 1.11 µM TE/g). However, when compared to quercetin (48.61 ± 2.00 μM TE/g), a well-known flavonoid with potent antioxidant properties, both fresh and dry key lime oils exhibit moderate antioxidant potential but remain lower in efficacy, which aligns with the expected antioxidant contributions of essential oils versus polyphenolic compounds (Table 3).

**TABLE 3:** ABTS radical scavenging potential of fresh and dry key lime essential oils expressed as Trolox Equivalent Antioxidant Capacity (µM Trolox equivalent (TE)/g).

Samples	ABTS (µM TE/g)
Fresh essential oil	37.76 ± 0.80
Dry essential oil	27.76 ± 1.11
Quercetin	48.61 ± 2.00

According to Gargouri et al., *Citrus paradisi* essential oil has high radical scavenging activity against ABTS free radicals. Our findings (37.76 μM TE/g for fresh key lime oil) are consistent with earlier studies indicating the antioxidant activity of citrus essential oils (Gargouri et al., 2021). Furthermore, Tundris et al. investigated the antioxidant activity of three citrus oils and found that they demonstrated a strong radical scavenging capacity. These findings highlight the potential of citrus essential oils as natural antioxidants, with *C. aurantifolia* essential oil exhibiting the highest activity among the studied varieties (Tundis et al., 2012).

The detected metabolites of key lime essential oils were stated to exhibit considerable pharmacological activity that is valuable in the skin care industry. Their antiaging and antioxidant properties were observed. These observed positive activities prompted us to investigate the anti-aging potential of both key limes’ essential oils using several *in vitro* and molecular docking studies. This activity may be ascribed to the percentage of α-Pinene and β-Pinene 2.79% and 10.88% in fresh key lime compared to dry key lime. Fraternale et al. reported that limonene and α-pinene, tested alone and in mixture, exhibited repressive activity against elastase and collagenase enzymes (Fraternale et al., 2019). Elgamal et al. reported that anti-aging effectiveness was directly related to antioxidant potential. The high percentage of sesquiterpene and oxygenated monoterpenes in fresh key lime essential oil, representing 6.82% and 13.51%, respectively, played an important role as an antioxidant agent and, therefore anti-aging (Elgamal et al., 2021). These reports determined that an increase in the free radical scavenging compounds in fresh key lime essential oil caused a rise in the anti-aging potential. All results concluded with the synergetic effect of the compounds of the essential oil. This fact about the role of synergetic action was extremely obvious in our data, where the fresh key lime essential oil demonstrated improved activity compared to dry key lime essential oil.

### 2.4 *In silico* studies

#### 2.4.1 Molecular docking

The essential oil’s promising inhibitory effect against collagenase and elastase prompted molecular docking research. Docking studies are carried out to determine the likely binding mechanism and pattern of drugs with potential targets. As previously stated, the major chemicals we found have been shown to have antiaging effects in various trials. Computational drug design research has shed light on the relevance of employing molecular docking in drug development to acquire important insights into molecules’ interactions and possible binding processes in protein binding sites (Singab et al., 2023). The key lime main metabolites were docked to the active sites of collagenase and elastase, and their binding modes, interaction energies, and 2D interaction diagrams are shown in Tables 4–6. The compounds’ interaction energies were in the range of −(6.8–7.9 and 7.1–7.9) Kcal/mol. The β- pinene exhibited the best docking scores with collagenase and elastase enzymes.

**TABLE 4:** Docked compounds’ interaction energies against the collagenase and elastase active sites.

Compound name	Docking scores (Kcal/mol)*	
	Collagenase	Elastase
β- Pinene	−7.9	−7.9
D- Limonene	−6.8	−7.6
γ- Terpinene	−7.3	−7.1

*Reference ligands energy was −7.8 and −8.9 kcal/mol, respectively.

*The RMSD from redocking of co-crystalized ligands was 0.74 and 0.83 Å.

**TABLE 5:** 2D interaction diagrams and the key lime major metabolite binding modes towards elastase.

Compound	Binding interactions	2D interaction diagram
β-pinene	Two H-bonding: (Ser 214)	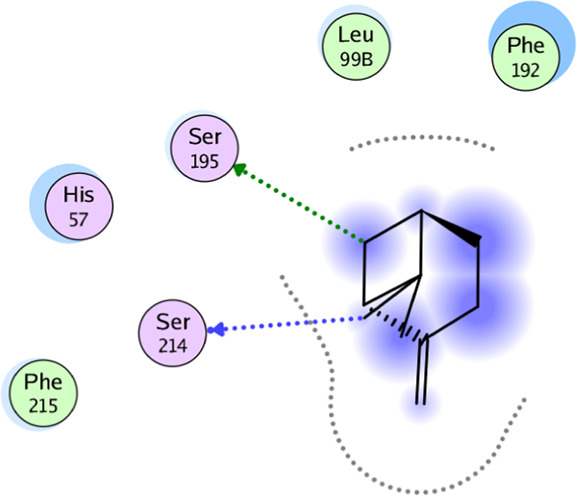
D-limonene	Two H-bonding: (Ser 214) One hydrophobic interaction: (His 57)	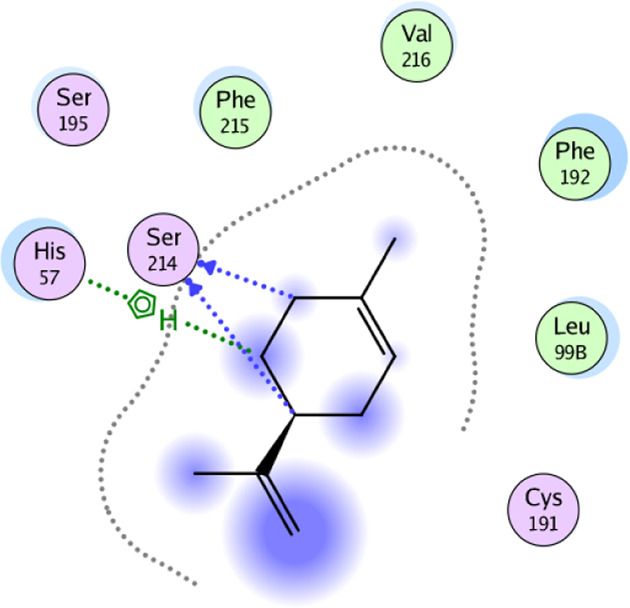
γ- Terpinene	One H-bonding: (Leu 167)	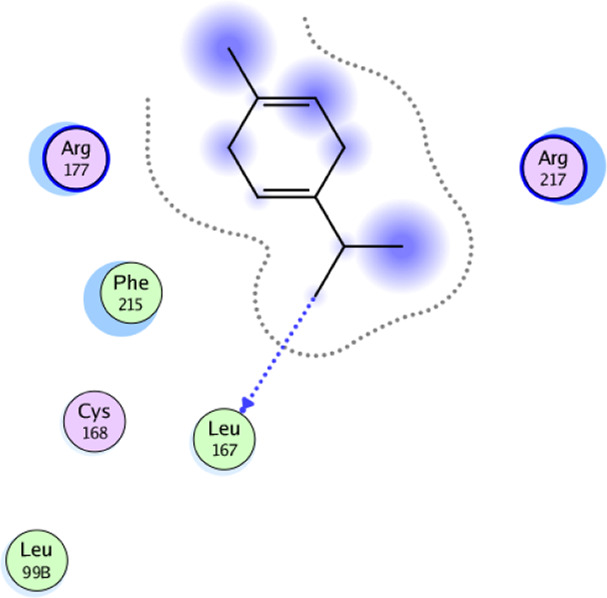
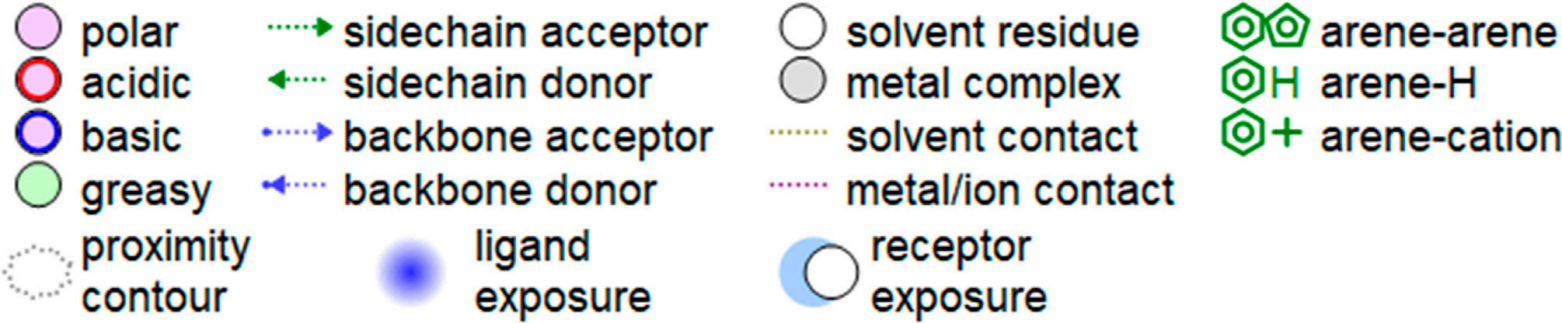		

**TABLE 6:** 2D interaction diagrams and binding modes of the key lime major metabolite towards collagenase.

Compound	Binding interactions	2D interaction diagram
β- Pinene	Three H-bonding: (Pro 238, Tyr 237, His 238) Two hydrophobic interactions: (His 238)	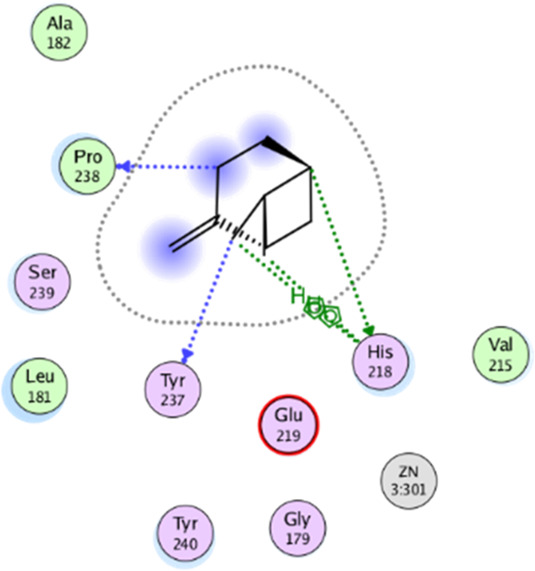
D- Limonene	Two H-bonding: (Asn 180, Glu 239) Two hydrophobic interactions: (His 218)	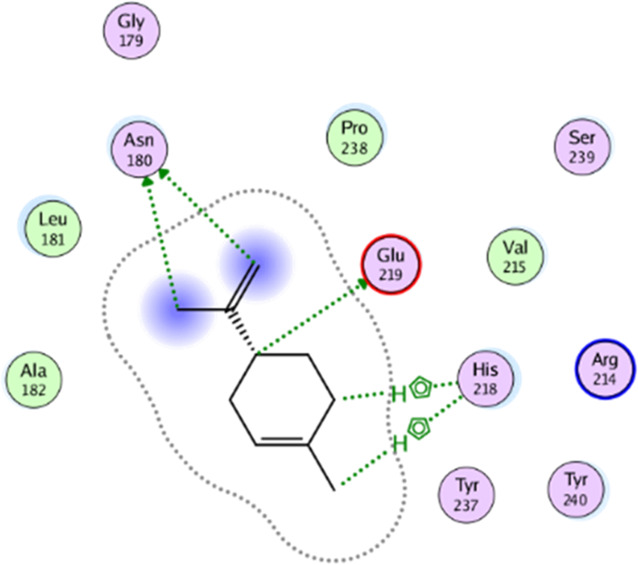
γ- Terpinene	Two H-bonding: (Asn 180, Ala 182, Ser 239)	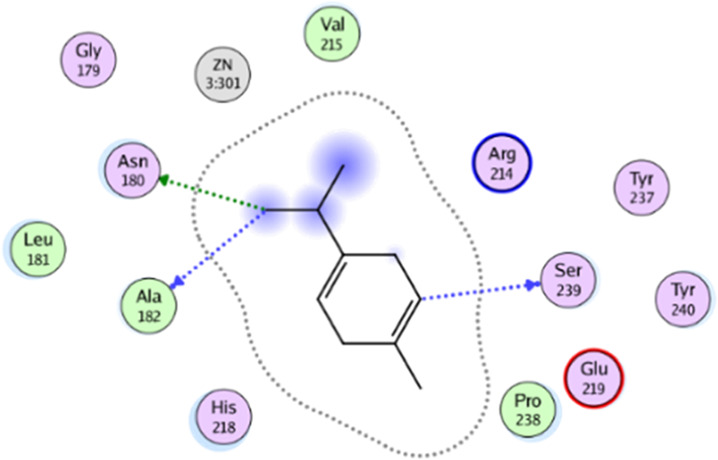
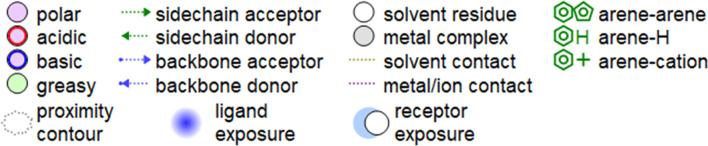		

#### 2.4.2 Molecular dynamics simulation (MDS)

Molecular dynamics (MD) simulations have offered significant insights into how drugs interact with their targets. These include precise assessments of ligand-target binding affinities, exploration of macromolecular behavior, and evaluation of how specific mutations influence drug resistance profiles. Within this framework, the complexes formed between B-Pinene and the enzymes collagenase and elastase were selected for MD simulation studies.

**FIGURE 1:**
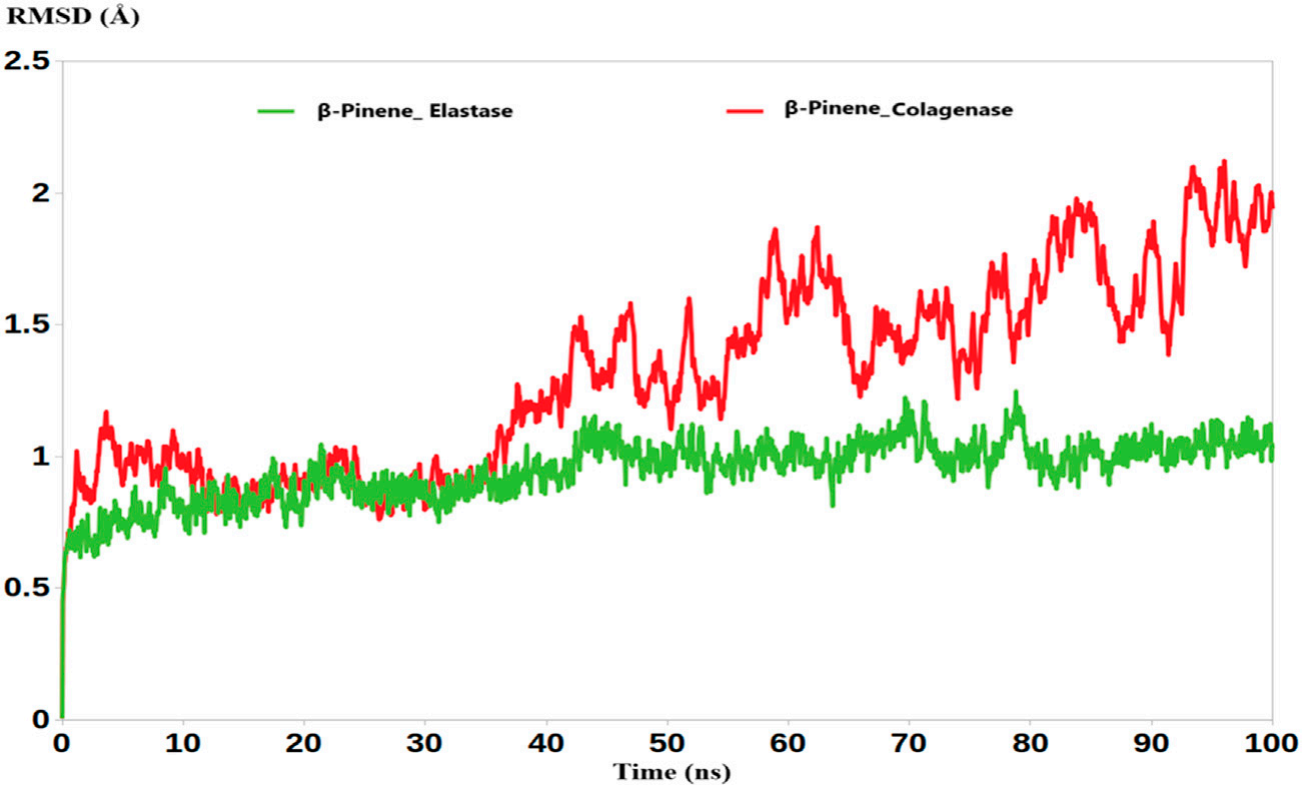
The RMSD analysis of B-Pinene bound to collagenase (red) and elastase (green).

Interestingly, the molecular dynamics (MD) simulations revealed that the average root mean square deviation (RMSD) values for the complexes of B-Pinene with collagenase and elastase were 1.91 Å and 1.34 Å, respectively (Figure 1). These relatively low RMSD values indicate minimal structural fluctuations of the protein-ligand complexes over the simulation time, suggesting that B-Pinene maintains a stable binding conformation within the active sites of both enzymes.

**FIGURE 2:**
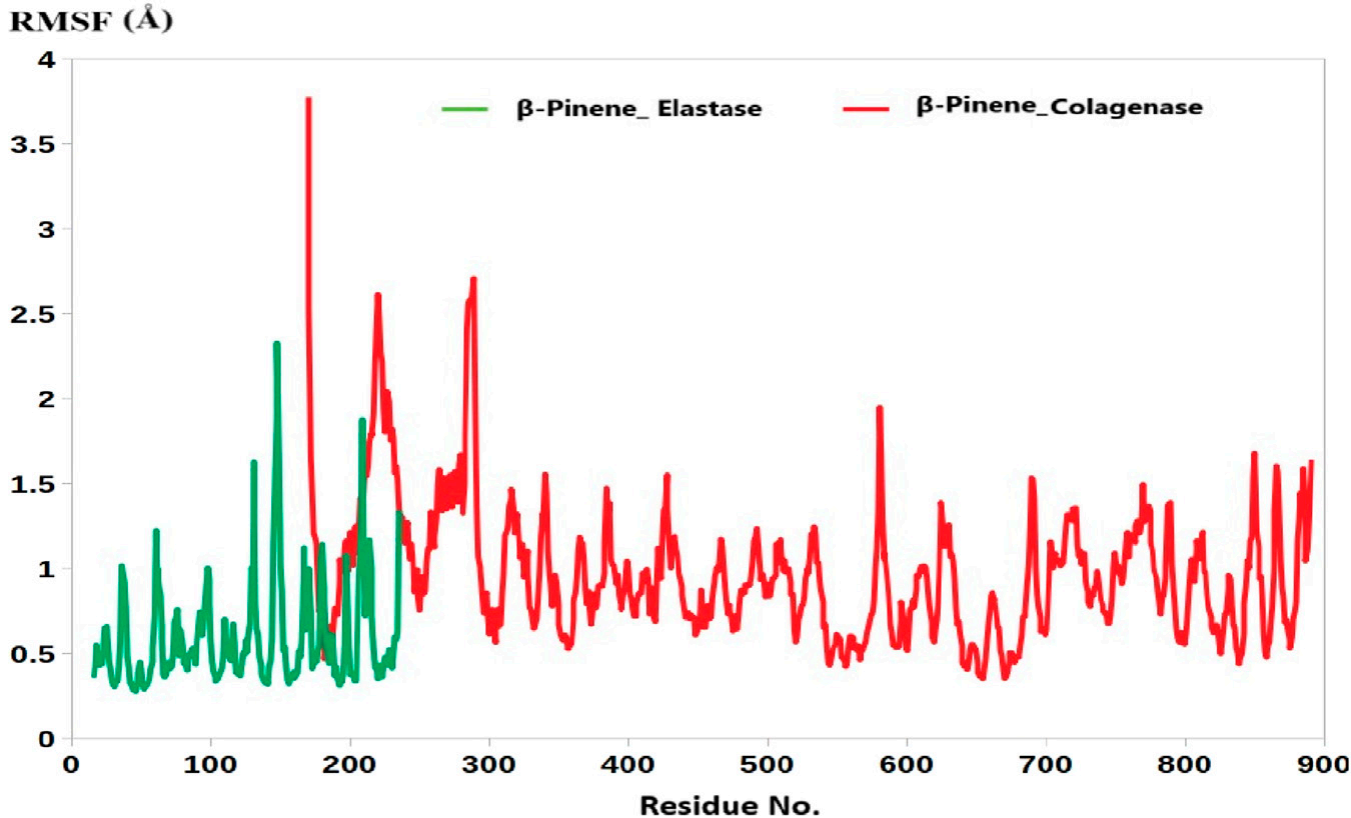
The RMSF analysis of B-Pinene bound to collagenase (red) and elastase (green).

In addition, the root mean square fluctuation (RMSF) analysis further supports this observation. The average RMSF value for the collagenase-B-Pinene complex was calculated at 1.77 Å, while that of the elastase-B-Pinene complex was slightly lower at 1.17 Å (Figure 2). RMSF measures the flexibility of individual amino acid residues throughout the simulation, and the low values observed here indicate that the residues in the binding regions of both enzymes remain relatively stable and are not highly flexible upon ligand binding.

Collectively, these results highlight the potential of B-Pinene to form stable and consistent interactions with both collagenase and elastase. The stable RMSD and RMSF profiles reinforce the reliability of our initial molecular docking results, confirming that the predicted binding poses are not only energetically favorable but also dynamically stable over time. These findings provide strong evidence supporting the use of B-Pinene as a potential inhibitor of these enzymes and validate the robustness of our computational docking and simulation workflow.

## 3 Conclusion

Finally, this study emphasizes the differences in chemical compositions between fresh and dry key lime (Citrus aurantifolia) essential oils, emphasizing the greater chemical complexity of fresh oil. Fresh oil contains more metabolites, notably oxygenated monoterpenes like α-terpineol and 4-terpinenol, indicating better bioactivity, especially for anti-aging uses. The found anti-elastase and anti-collagenase actions, as validated by molecular docking and *in vitro* experiments, suggest that fresh key lime oil has potential as a natural cosmetic product. However, because these findings are based on *in vitro* and *in silico* techniques, additional *in vivo* research and clinical validation are required to validate efficacy and safety. Future research should investigate the stability, formulation potential, and long-term production of fresh vs. dry essential oils for industrial uses, ensuring their viability in the skincare and pharmaceutical industries.

## 4 Methods

### 4.1 Plant material

From the Egyptian market in Cairo, fifth settlement, fresh *Citrus aurantifolia* were collected (July, 2024) and authenticated by Dr. Therese Labib Yousef, Director of Herbarium, Consultant of botanical gardens and National Gene Bank. Voucher number (L20-12) was preserved at Pharmacognosy department, Future University in Egypt, Faculty of pharmacy.

### 4.2 Essential oil isolation

Three kilograms of fruit were washed using flowing water and then toweled to eliminate any surface dust. The clean fruits were divided into two-halves. The first half of key lime was subjected to shade drying for 3 weeks till complete drying (same weight for 3 consecutive days) (temperature range 30°C–36°C with 25%–40% humidity, good airflow and frequent rotation to avoid mold growth). Just before hydro-distillation, fresh and dry fruits were grounded for 4 h (Ngo et al., 2020; Veloso et al., 2020) in a Clevenger apparatus to separate the essential oils. Over anhydrous Na_2_SO_4_, the oils were dried and kept in a shaded brown glass container in the refrigerator (4°C) till analysis. Based on the mentioned equation, the yield was calculated:

$$\text{Yield of essential oil }\left(\%\;v/w\right)=\left[\text{essential oil volume }\left(\text{mL}\right)/\text{ plant Weight }\left(g\right)\right]\times 100$$

### 4.3 Gas chromatography–mass spectrometer analysis

Shimadzu Gas chromatography–Mass spectrometer -QP2010 (Tokyo, Japan), Rtx-5MS column (30 m × 0.25 mm i.d. × 0.25 µm film thickness) and a split–splitless injector as per previous procedures (Elkhawas et al., 2023). Isothermally, the starting temperature of the column was 45°C for 2 min, then programmed to 300°C with a rate of 5°C/min. Helium was used as a carrier gas with a flow rate of 1.41 mL/min and an injection volume 1 μL was employed in split mode at a split ratio of 1:15. The ionization voltage was 60 mA of 70 eV, and the ion source was 200°C. Compounds were identified based on their Kovats index (KI) and cross-referenced with NIST, Adams, and relevant literature data.

### 4.4 *In Vitro* anti-ageing evaluation

#### 4.4.1 Anti-collagenase analyse

The anti-collagenase test was achieved colorimetric (ab 196999) as per the procedure stated by (Thring et al., 2009; Imran et al., 2023) with minimal changes. Collagenase from *Clostridium* histolyticum (ChC—EC.3.4.23.3) was dissolved in the 50 mM Tricine buffer to 0.8 unit/mL initial concentration. N-[3-(2-furyl) acryloyl]-Leu–Gly–Pro–Ala (FALGPA) was used as a substrate and dissolved in Tricine buffer to a final concentration of 2 mM. The absorbance values were measured at 490 nm. A positive control (piroxicam) was used.

#### 4.4.2 Anti-elastase assay

The anti-elastase test was achieved spectrophotometrically as previously described by Kim et al. (Imran et al., 2023; Kim et al., 2004) with minimal changes. Porcine pancreatic elastase was dissolved in sterile water to prepare a stock solution with a concentration of 3.33 mg/mL. N-Succinyl-Ala–Ala–Ala–p-nitroanilide (AAAPVN) was used as a substrate and dissolved in Tris-HCl buffer at a concentration of 1.6 mM. Daidzein was used as positive control. The absorbance was measured at 400 nm.

#### 4.4.3 2,2′-azino-bis(3-ethylbenzothiazoline-6-sulfonic acid (ABTS) assay

The assay was performed as previously reported by Floegel et al. (2011). The ABTS capacity of both oils was calculated as Trolox equivalent antioxidant capacity (TEAC) and expressed as µM TE/g.

### 4.5 Statistical analysis

The assays were performed in triplicates, and the values are presented as mean ± SD. For *in vitro* anti-collagenase, and anti-elastase activities, the (IC_50_) was predicted from the graph plots of the dose–response curves at each sample concentration by Graph Pad Prism software (San Diego, CA, United States) and statistical analysis was carried out using one way ANOVA.

### 4.6 *In silico* studies

#### 4.6.1 Molecular docking

Molecular docking of the major metabolites of key lime was accomplished on the crystal structure of collagenase and elastase enzymes, recovered as co-crystallized with a ligand from the protein data bank (PDB ID: 1W3Y, and 1b0f). Docking exploration was conducted using Accelrys Inc.’s Discovery Studio 4.5 software in San Diego, California, United States. Using the default program protocol, ligands and enzymes were initially created. The active binding site of collagenase and elastase enzymes was then determined by analyzing the binding mode of the co-crystallized reported ligand active conformer. The structures of the compounds under consideration were docked into the enzyme’s active binding site pocket using the C-Docker technique. The program provided 2D binding diagrams and C-Docker binding energies for evaluation (Elkhawas et al., 2023; El-Nashar et al., 2023; Ramlal et al., 2024).

#### 4.6.2 Molecular dynamics simulation (MDS)

The two molecular dynamics of B-Pinene in complex with collagenase and elastase were performed using Gromacs and Acypype. The typical workflow of the reported methodology was applied for 100 ns of molecular simulation (Elgohary et al., 2025; El Hassab et al., 2025; Majrashi et al., 2024).

## Data Availability

The original contributions presented in the study are included in the article/supplementary material, further inquiries can be directed to the corresponding authors.
